# Editors’ Pick: milk sugar, migration and pastoralism in Africa

**DOI:** 10.1186/2041-2223-5-5

**Published:** 2014-03-31

**Authors:** Antti Sajantila

**Affiliations:** 1 Department of Forensic Medicine, Hjelt Institute, University of Helsinki, P.O.Box 40, 00014 Helsinki, Finland; 2 Institute of Applied Genetics, Department of Molecular and Medical Genetics, University of North Texas Health Science Center, 3500 Camp Bowie Blvd, Fort Worth, TX US 76107, USA

## 

Lactose persistence (LP), the ability to digest milk sugar (lactose), is one of the best examples of selection-based evolutionary change in humans from milk-drinking cultures. LP has been documented in populations with northern European ancestry and those originating in Central Asia, Middle East, the Arabian Peninsula, and Africa. Based on molecular genetic studies, it has been estimated that the selective change in the ability to digest lactose beyond childhood occurred within approximately the past 5,000-10,000 years [1]. The estimation is consistent with an advantage to LP in dairy farming-based subsistence, which came to prominence in certain geographical areas around the same time.

The important enzyme in the breakdown of milk sugar, *lactase* (or more specifically lactase-phlorizin hydrolase (LPH)), is coded by the LCT gene. The molecular genetic data reported in recent years show several interesting aspects of LP. The first genetic variation described for European LP was not in the LCT gene, but in the in the intron 13 of the MCM6 gene, residing ~14 kb upstream of MCM6 [2]. However, it soon was realized that the MCM6 intron 13 T-13910 allele, which explained most LP in Europe, was absent in Africa [3,4]. Instead, three other SNPs in the same intron, C-14010, G-13907 and G-13915, were associated with lactase persistence in Africa. Also, these SNPs originated on different haplotype backgrounds from the European T-13910 allele and from each other providing a major model of convergent evolution in LP due to strong selective pressure [3,5].

To date, several SNPs associated with LP are known. In Europe, in addition to MCM6 intron 13 T-13910, intron 9 G-22018 is significantly associated with the LP trait. In Africa and the Arabian Peninsula, four SNPs (those above and G-14009), which are all located within 100 bp of T-13910 have been shown to be associated with the LP predominantly in pastoralist populations. Those variants do not, however, account for all the phenotypic variance of LP in Africa, implying that additional genetic variation may play a role.

A new study by Ranciaro *et al*. in *American Journal of Human Genetics*[6] sought out new genetic variants associated with LP in Africa. They studied 819 individuals from 63 African populations, and 154 individuals from non-African populations from Europe, the Middle East and Asia. The authors sequenced MCM6 gene introns 9 and 13 and ~2 kb of the LCT gene promoter region in these samples. Additionally, in an effort to reconstruct the origin and spread of LP-associated alleles in Africa, four microsatellites were genotyped in a ~198 kb region in a subset of 252 individuals. The participants’ LP status was tested using a standardized blood test that tracks lactose digestion.

With the new larger data set, and sampling from outside Africa, the authors confirmed the earlier observed association between the LP trait in Africa and three variants in intron 13, and they also found two additional LP-associated SNPs, one in intron 13 (G-12962) and one in the LCT promoter region (T-956). Further functional studies are naturally needed to prove whether these new SNPs are causative or just merely in linkage disequilibrium with the earlier identified variants. Ranciaro *et al*. [6] then used allele frequency and long-range linkage disequilibrium based neutrality tests to detect any signatures of selection. They found evidence for recent positive selection in eastern African populations and the Fulani from central Africa.

While looking for the new variants for LP within Africa, the study by Ranciaro *et al*. [6] also considered that if LP patterns coincide with the rise in pastoralism, the LP-related alleles found in present-day populations should reveal past migrations of those populations. In order to do that, they analyzed a subset of 252 study participants using four microsatellites and combined these with the SNPs to create haplotypes. The haplotype analysis has some interesting insights about the migration of pastoralism groups in Africa. For example, the haplotype analysis supported an eastern African origin (Kenya and Tanzania) of the C-14010 LP-associated mutation now found in southern Africa in Bantu-speaking Xhosa and the hunter-gatherer San population. Also, the presence of Middle Eastern origin G-13915 allele in North African populations suggests migration between these regions. Interestingly, the data are supported by known historical interactions between these populations. Some of the data in Ranciaro *et al*. [6] is in agreement with earlier findings of distinct genetic LP variants that have arisen in various parts of Africa through convergent evolution. For example, the G-13907 was almost exclusively restricted to northern Kenya, Ethiopia and northern Sudan, as reported earlier. Last but not least, the authors also detected the traditionally European LP haplotype background in some West-and North-Central African populations. But, even with these new data from Ranciaro *et al*. [6], the genetic variants found and analyzed in various populations to date, are far from fully explaining LP in Africa.

## Competing interests

The author declares that they have no competing interests.
